# Prediction of BRAF mutation status in glioblastoma multiforme by preoperative ring enhancement appearances on MRI

**DOI:** 10.3389/fonc.2022.937345

**Published:** 2022-08-08

**Authors:** Xiaomin Cai, Zheng Chen, Bowen Chang, Ming Tu, Shiting Li, Xuhui Wang, Ming Chen

**Affiliations:** ^1^ Department of Neurosurgery, Xinhua Hospital of Shanghai Jiaotong University School of Medicine, Shanghai, China; ^2^ Department of Neurosurgery, The First Affiliated Hospital of USTC, Division of Life Sciences and Medicine, University of Science and Technology of China, Hefei, China; ^3^ Department of Neurosurgery, The First Affiliated Hospital of Wenzhou Medical University, Wenzhou, China; ^4^ Department of Neurosurgery, Xinhua Hospital of Shanghai Jiaotong University School of Medicine, the Cranial Nerve Disease Center of Shanghai Jiaotong University, Shanghai, China; ^5^ Department of Neurosurgery, Xinhua Hospital of Shanghai Jiaotong University School of Medicine, Chongming Branch, Shanghai, China

**Keywords:** Glioblastoma multiforme, BRAF, Ring enhancement, MRI, Nomogram

## Abstract

**Background:**

Ring enhancement on magnetic resonance imaging (MRI) is an important characteristic of GBM. Though patients suffering from glioblastoma multiforme (GBM) with BRAF mutation (MUT BRAF) in V600E benefit from BRAF-targeted inhibitors, the relationship between ring enhancement and MUT BRAF remains elusive. The purpose of this study was to investigate the relationship between BRAF mutation status and the appearance of ring enhancement so as to guide preoperative targeted therapy for MUT BRAF GBM.

**Methods:**

Patient’s population, clinical data and characteristic ring enhancement appearances on MRI were compared between GBM with MUT BRAF and GBM with WT BRAF. A receiver operating characteristic (ROC) curve analysis was performed to evaluate the differential diagnostic significance. A nomogram was developed to predict the mutation status of BRAF. Moreover, all the variables were re-analyzed between epithelioid GBM (E-GBM) with or without MUT BRAF.

**Results:**

Compared to GBM with WT BRAF, GBM with MUT BRAF had specific ring enhancement appearances with multiple rings, multiple located lobes, regular shape of ring, uniform thickness of ring and smaller diameter of ring. Area under the curve (AUC) of all the variables’ combination was 0.929. The nomogram was developed and validated. The re-analyzed results between E-GBM with or without MUT BRAF were similar to these above. AUC of the combination of quantity of ring, quantity of located lobe and shape of ring was 0.962.

**Conclusion:**

The characteristic ring enhancement appearances of GBM may play an important role in predicting BRAF mutation status preoperatively, especially in E-GBM. Further study with larger cases may provide more evidences to guide the pretreatment of targeted medicine for GBM patients with MUT BRAF in future.

## Introduction

Glioblastoma multiforme (GBM) is a common malignant tumor in central nervous system (CNS). Its incidence is about 5.26/100,000 and has been increasing yearly. At present, maximal surgical resection complemented by concurrent radiotherapy and chemotherapy is still the first-line treatment for GBM in clinic. Although lots of efforts are made, the prognosis of most patients with GBM is still dismal. An improved method is urgently needed (1–5).

Many researches about special genes in GBM have been reported previously (6). For instance, GBM with mutant Isocitrate dehydrogenase 1 (IDH1) indicates better prognosis than wild-type IDH1 GBM (7–9). The methylation of O6-methylguanine-DNA-methyltransferase (MGMT) indicates better chemotherapy effects (10). Recently, it was reported that B-Raf (BRAF) is a prognostic marker in glioma (11–17). Clinical trials showed that those GBM patients with mutated BRAF (MUT BRAF) in V600E benefited from BRAF-targeted inhibitors (18, 19). However, whether a GBM had BRAF mutation or not must be confirmed by postoperative pathological examination. Because these processes usually take a long time, some patients hence missed the best time for treatment. Thus, if whether BRAF is mutated or not could be predicted *via* noninvasive methods preoperatively, the follow-up treatment and molecularly-targeted agents would be applied in a timely manner. It is worth noting that GBM often presents ring enhancement on magnetic resonance imaging (MRI). This special image appearance may contain some potential information about GBM’s biological characteristics which is closely associated with genes.

Therefore, the purpose of this study was to investigate the relationship between mutated status of BRAF and the appearances of ring enhancement on MRI in GBM. These results may help us to predict whether BRAF is mutated or not *via* noninvasive method preoperatively and guide timely follow-up treatment with targeted drugs, such as BRAF inhibitors in the future.

## Methods

### Patient population and tissue samples

This study was approved by the Ethics Committee of Xinhua Hospital, School of Medicine, Shanghai Jiaotong University. All patients (or their guardians) enrolled in the study had signed informed consents. Medical records of patients with a diagnosis of primary GBM operated at Xinhua Hospital of Shanghai Jiaotong University and The First Affiliated Hospital of Wenzhou Medical University between January 2016 and December 2020 were collected and retrospectively analyzed. Records included in the final analysis met the following criteria: 1) The patient had not undergone surgery, radiotherapy and chemotherapy previously; 2) Complete preoperative MRI data including both non-enhanced and contrast-enhanced sequences; 3) Formalin-fixed and paraffin-embedded (FFPE) tumor tissues; 4) Histopathological diagnosis of primary GBM reviewed and confirmed by 2 experienced neuropathologists in consensus according to WHO 2016 criteria. The exclusion criteria were incomplete preoperative MRI data and insufficient FFPE tissues for analysis. Informed consent was obtained from eligible patients.

### Clinical data collection

Electronic medical records were reviewed for patient demographic characteristics and presenting symptoms. The patients’ chief complaints and associated symptoms were classified into intracranial hypertension, hemiplegia and speech vague. Therapeutic strategies were established according to the above information combined with radiological data, and all patients in this study received surgical treatment.

### Polymerase chain reaction and mutation analysis

DNA was extracted from FFPE tissue as follows. Tissues from the representative area of 90% tumor content were scraped off deparaffinized sections into tubes and treated in 10-mM Tris-HCl buffer with proteinase K at 55°C for 12 hours and then at 98°C for 10 minutes. The cell lysate was centrifuged, and supernatant was collected for polymerase chain reaction (PCR). Primer pairs were used for gene amplification of BRAF V600E. Forward: 5’-TGCTTGCTCTGATAGGAAAATG-3’. Reverse: 5’-CCACAAAATGGATCCAGACA-3’. The detailed PCR procedure was the same as previous literature.

### Imaging data acquisition and analysis

MRI images were obtained with a 3.0-T MRI scanner. Imaging parameters of axial, sagittal, and coronal T1-weighted sequences were as follows: TE 9.1 msec, TR 2000 msec, FOV 20 × 20 cm, slice thickness 2.5 mm, and matrix size 256 × 256. Enhanced sagittal and coronal T1-weighted images were acquired with the same parameters after Gd-DTPA injection (0.2 ml/kg). Two neuroradiologists first reviewed all MRI images (blinded to patient identity and clinical characteristics, including mutation status of BRAF) and then resolved their discrepancies in consensus. Quantitative measurements were made on a picture archiving and communication system (PACS). The characteristic ring enhancement appearances on MRI included location, side, shape, quantity, closed or open ring, thickness of ring margin, maximal diameter, interior signal and edema ratio. Main location meant the lobes where more than 50% of the tumor volume was located. Side of lesion referred to the side where more than 50% of the tumor volume was located. Located lobules meant the number of lobes, which the ring enhancement on MRI is located in. And, brain lobes are divided according to their anatomical locations. Ring enhancement maximal diameter (size) was calculated on all T1 enhancing MRI slices, and the largest value was recorded and identified as maximal diameter of ring. Closed ring meant that the enhanced ring was complete and short of any chips on all levels of T1 enhancement MRI images. The shape of ring was evaluated on T1 enhancement images and described as regular or irregular. Regular shape meant circle or oval. The thickness of ring margin was classified into 2 categories including uniform and nonuniform. We first chose one MRI slice which showed the largest diameter of ring enhancement. Then, the maximal and minimal thickness of ring were measured at this MRI slice. Ring was regarded as uniform when the difference between maximal and minimal thickness was no more than 50%, vice nonuniform. Compared with the signal of cortex, the interior signal of ring on T1 enhancement image was described as hyper-, iso-, or hypointense. Edema ratio referred to the ratio of maximal diameter of tumor on T1 enhancing MRI to maximal diameter of tumor edema on T2-weighted MRI.

### Statistical analysis

Statistical analyses were performed by using SPSS software version 25.0. Initially, normal distribution of the variables was analyzed by the Kolmogorov-Smirnov test. Normally distributed data were analyzed by 2-tailed Student t test or one-way analysis of variance. For nonparametric data, Mann-Whitney U test was used for comparisons between groups. The diagnostic performances of all the variables were assessed by values of AUC obtained from the ROC curve. After *post hoc* analysis, a cutoff for the abnormal score was determined by the value corresponding to maximal sum of sensitivity and specificity. Logistic regression multivariate analysis of variance was also used to explore related risk factors, which were then used to develop the regression model and transformed into a nomogram. However, since the mutation of BRAF was closely related to E-GBM, the statistical analysis mentioned above was conducted again between E-GBM with MUT BRAF and E-GBM with wild-type BRAF (WT BRAF). A 2-tailed *p* value < 0.05 was considered statistically significant.

## Results

### Clinical and pathological findings

Forty-four patients with primary GBM were included in this study. 15 of 44 cases exhibited the BRAF V600E mutation (13 of 19 E-GBMs, 1 of 2 gliosarcomas, 0 of 1 giant cell glioblastoma, 1 of 22 conventional GBMs). The GBM patients’ mean age with MUT BRAF was 59.33 ± 1.29, while the WT BRAF group was 58.52 ± 16.07. Intracranial hypertension symptoms were the most common complaint for patients with WT BRAF tumors (16 of 29 cases). The distribution of clinical symptoms in patients with MUT BRAF tumors was equal (**Table 1**).

**Table 1:** Demographics and clinical characteristics of GBM cases stratified by BRAF mutation status.

Characteristics	MUT BRAF (n=15)	WT BRAF (n=29)	*p* Value
Clinical features			
Mean age (years)	59.33±1.29	58.52±16.07	0.291
Sex			0.521
Male	10	22	
Female	5	7	
Main symptoms			0.080
Intracranial hypertension	5	16	
Hemiplegia	5	10	
Speech vague	5	3	
MRI features			
Main locations			0.182
Frontal	5	9	
Temporal	10	11	
Insular	0	2	
Parietal	0	2	
Occipital	0	1	
Brainstem	0	1	
Corpus callosum	0	3	
Side of lesion			0.766
Left	5	11	
Right	10	18	
Quantity of ring			0.002
Single	3	20	
Multiple	12	9	
Quantity of located lobe			0.025
Single	5	20	
Multiple	10	9	
Closed ring			0.472
Yes	15	28	
No	0	1	
Shape of ring			0.009
Regular	12	11	
Irregular	3	18	
Thickness of ring			0.003
Uniform	10	6	
Nonuniform	5	23	
Maximal thickness of ring (cm)	0.40±0.14	0.68±0.44	0.106
Maximal diameter of ring (cm)	4.32±0.80	5.09±1.44	0.016
Maximal diameter of edema (cm)	8.39±2.12	7.76±0.67	0.094
Edema ratio	0.57±0.13	0.64±0.23	0.249
Inferior signal of ring			0.099
Low-intensive	5	7	
Iso-intensive	10	14	
High-intensive	0	8	

Values are numbers of cases or patients unless otherwise indicated. Mean values are presented with SDs.

### MRI findings

Most GBMs with MUT BRAF or WT BRAF were located in temporal lobe and in right side. The mean maximum diameter of ring in the MUT BRAF tumors was 43.2 mm, while the WT BRAF tumors was 50.9 mm (*p* = 0.016). Most MUT BRAF tumors exhibited a regular shape, while BRAF WT tumors exhibited irregular shapes (*p* = 0.009). The quantity of ring of MUT BRAF tumor tended to be multiple which was different from BRAF WT tumor (*p* = 0.002). Most MUT BRAF tumors located in multiple lobes while another group tended to be located in single lobe (*p* = 0.025). Almost all of the enhanced rings were closed. Most MUT BRAF tumors were with uniform thickness of ring, while most WT BRAF tumors were with nonuniform thickness of ring (*p* =0.003). There were not significant differences in maximal thickness of ring, maximal diameter of edema, edema ratio, inferior signal of ring between MUT BRAF and WT BRAF tumors (**Table 1**).

### Diagnostic criteria for GBM with MUT BRAF

**Figure 1:**
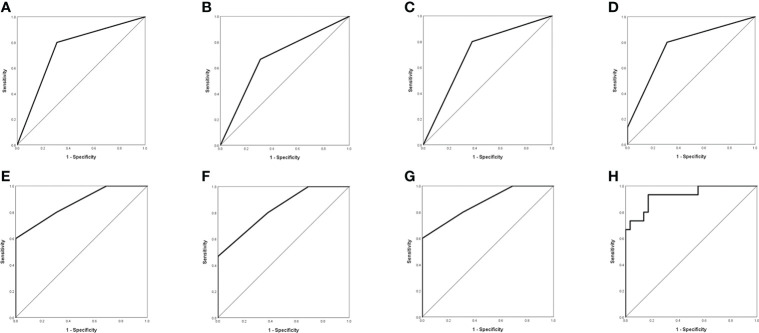
Evaluation of differential diagnosis efficacy for MUT BRAF and WT BRAF in GBM. **(A)**: The AUC for quantity of ring was 0.745 (95% CI 0.590-0.900); **(B)**: The AUC for quantity of located lobe was 0.678 (95% CI 0.508-0.849); **(C)**: The AUC for shape of ring was 0.710 (95% CI 0.550-0.871); **(D)**: The AUC for quantity of ring + quantity of located lobe was 0.766 (95% CI 0.613-0.918); **(E)**: The AUC for quantity of ring + shape of ring was 0.869 (95% CI 0.753-0.985); **(F)**: The AUC for quantity of located lobe + shape of ring was 0.830 (95% CI 0.702-0.957); **(G)**: The AUC for quantity of ring + quantity of located lobe + shape of ring was 0.869 (95% CI 0.753-0.985); **(H)**: The AUC for quantity of ring + quantity of located lobe + shape of ring + thickness of ring +maximal diameter of ring was 0.929 (95% CI 0.846-1.000).

Based on comparisons between WT BRAF and MUT BRAF GBM groups, we suggested MRI characteristics of MUT BRAF GBM as multiple rings, multiple located lobes, regular shape of ring, uniform thickness of ring and smaller diameter of ring (**Table 2**). **Figure 1** showed the diagnostic value of preoperative radiological appearance for GBM patients with MUT BRAF. Their corresponding AUC values with 95% confidence interval (CI) are presented in **Table 3**. Only the AUC values of quantity of ring, quantity of located lobe and shape of ring were over 0.6. The highest AUC value of them was 0.745. Thus, ROC curves were drawn to analyze the validity of diagnostic criteria using various combinations of these features. For diagnostic criteria based on the presence of quantity of ring and shape of ring, the AUC was 0.869. For diagnostic criteria based on the presence of quantity of ring, quantity of located lobe and shape of ring, the AUC was also 0.896. For criteria based on the presence of all the five features in **Table 2**, the area under the ROC curve was 0.929 (**Table 3**).

**Table 2:** Diagnostic criteria of GBM with MUT BRAF.

Parameters	Manifestations on MRI
Multiple	Quantity of ring
Multiple	Quantity of located lobe
Regular	Shape of ring
Uniform	Thickness of ring
Smaller diameter	Maximal diameter of ring

**Table 3:** Evaluation of differential diagnosis efficacy for MUT BRAF and WT BRAF in GBM.

	MUT BRAF vs WT BRAF			
	AUC (95% CI)	Sensitivity	Specificity	Cutoff
Quantity of ring	0.745 (0.590-0.900)	0.800	0.690	0.500
Quantity of located lobe	0.678 (0.508-0.849)	0.667	0.690	0.500
Shape of ring	0.710 (0.550-0.871)	0.800	0.621	0.500
Thickness of ring	0.270 (0.105-0.435)	0.000	1.000	2.000
Maximal diameter of ring	0.276 (0.124-0.428)	1.000	0.138	3.090
Quantity of ring + Quantity of located lobe	0.766 (0.613-0.918)	0.800	0.690	0.328
Quantity of ring + Shape of ring	0.869 (0.753-0.985)	0.800	0.690	0.232
Quantity of located lobe + Shape of ring	0.830 (0.702-0.957)	0.467	1.000	0.656
Quantity of ring + Quantity of located lobe + Shape of ring	0.869 (0.753-0.985)	0.600	1.000	0.625
Quantity of ring + Quantity of located lobe + Shape of ring + Thickness of ring + Maximal diameter of ring	0.929 (0.846-1.000)	0.933	0.828	0.137

AUC, area under curve.

### Development and validation of the nomogram

**Figure 2:**
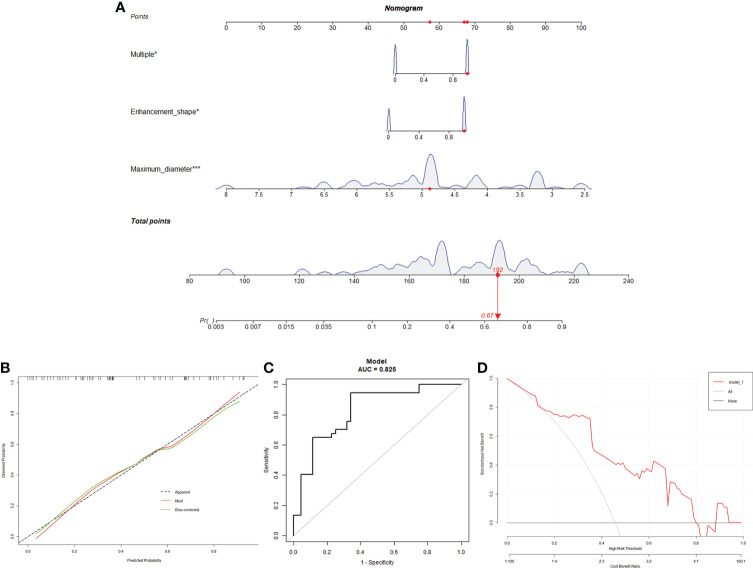
A Nomogram for predicting the mutation status of BRAF. **(A)**: MRI manifestation corresponds to a specific point by drawing a line straight upward to the points axis. After the sum of the points is located on the total points axis, the sum represents the probability of MUT BRAF. **(B)**: The calibration curve of the model in line with the agreement between predicted and observed outcomes. **(C)**: ROC curve was made to evaluate the discriminating capability of the nomogram. **(D)**: The DCA was drawn with the net benefit rate as the ordinate and the high-risk threshold as the abscissa.

Based on the logistic regression multivariate analysis results (**Supplementary Table 1**), we developed a nomogram model to predict the mutation status of BRAF (**Figure 2**). Each MRI manifestation corresponded to a specific score, and a linear point axis was plotted to calculate the total score, which corresponded to a higher probability of MUT BRAF. As shown in **Figure 2**, the prediction model had superior discriminant ability, and the area under ROC curve was 0.825 (95% CI: 0.7348, 0.9149). Furthermore, the established model was verified internally using the bootstrap verification method, with a C-index of 0.80088. Calibration curves were also generated. Results in **Figure 2** showed good consistency between the predictions and observations. Additionally, the decision curve analysis (DCA) was drawn with the net benefit rate as the ordinate and the high-risk threshold as the abscissa, with the high-risk threshold set to (0.0, 0.8).

### Re-analyzed results of E-GBM

**Figure 3:**
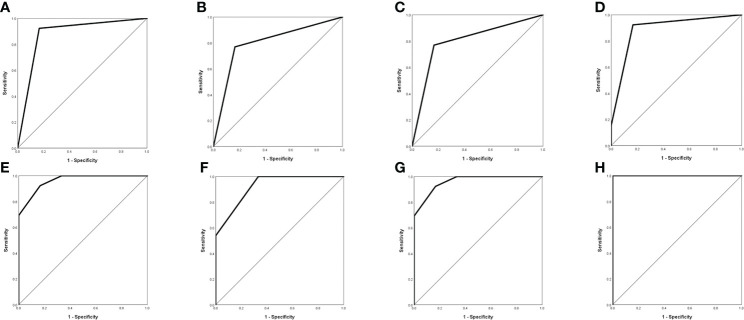
Evaluation of differential diagnosis efficacy for MUT BRAF and WT BRAF in E-GBM. **(A)**: The AUC for quantity of ring was 0.878 (95% CI 0.681-1.000); **(B)**: The AUC for quantity of located lobe was 0.801 (95% CI 0.578-1.000); **(C)**: The AUC for shape of ring was 0.801 (95% CI 0.578-1.000); **(D)**: The AUC for quantity of ring + quantity of located lobe was 0.891 (95% CI 0.715-1.000); **(E)**: The AUC for quantity of ring + shape of ring was 0.962 (95% CI 0.880-1.000); **(F)**: The AUC for quantity of located lobe + shape of ring was 0.923 (95% CI 0.794-1.000); **(G)**: The AUC for quantity of ring + quantity of located lobe + shape of ring was 0.962 (95% CI 0.880-1.000); **(H)**: The AUC for quantity of ring + quantity of located lobe + shape of ring + thickness of ring + maximal diameter of ring + maximal thickness of ring was 1.000 (95% CI 1.000-1.000).

It was similar to the previous results that E-GBMs with MUT BRAF tended to have multiple rings, multiple located lobes, regular shape of ring, uniform thickness of ring, smaller diameter of ring and smaller thickness of ring when compared with E-GBMs with WT BRAF (**Tables 4**, **5**). The AUC showed that quantity of ring (AUC: 0.878; 95% CI 0.681-1.000), quantity of located lobe (AUC: 0.801; 95% CI 0.578-1.000) and shape of ring (AUC: 0.801; 95% CI 0.578–1.000) had greater predictive value for MUT BRAF than the other 3 variables. When combined together, their AUC was 0.962 (95% CI 0.880–1.000) (**Figure 3**; **Table 6**).

**Table 4:** Demographics and clinical characteristics of E-GBM cases stratified by BRAF mutation status.

Characteristics	E-GBM with MUT BRAF (n=13)	E-GBM with WT BRAF (n=6)	*p* Value
Clinical features			
Mean age (years old)	59.23±1.30	66.67±9.81	0.246
Sex			0.913
Male	9	4	
Female	4	2	
Main symptoms			0.852
Intracranial hypertension	5	2	
Hemiplegia	4	3	
Speech vague	4	1	
MRI features			
Main locations			0.196
Frontal	5	1	
Temporal	8	4	
Insular	0	0	
Parietal	0	0	
Occipital	0	1	
Brainstem	0	0	
Corpus callosum	0	0	
Side of lesion			0.265
Left	5	4	
Right	8	2	
Quantity of ring			0.001
Single	1	5	
Multiple	12	1	
Quantity of located lobe			0.016
Single	3	5	
Multiple	10	1	
Closed ring			1.000
Yes	13	6	
No	0	0	
Shape of ring			0.016
Regular	10	1	
Irregular	3	5	
Thickness of ring			0.006
Uniform	9	0	
Nonuniform	4	6	
Maximal thickness of ring (cm)	0.39±0.14	1.06±0.41	0.002
Maximal diameter of ring (cm)	4.36±0.79	5.05±1.26	0.020
Maximal diameter of edema (cm)	7.69±0.69	9.25±1.94	0.108
Edema ratio	0.58±0.14	0.57±0.20	0.858
Inferior signal of ring			0.281
Low-intensive	4	1	
Iso-intensive	9	4	
High-intensive	0	1	

Values are numbers of cases or patients unless otherwise indicated. Mean values are presented with SDs.

**Table 5:** Diagnostic criteria of E-GBM with MUT BRAF.

Parameters	Manifestations on MRI
Multiple	Quantity of ring
Multiple	Quantity of located lobe
Regular	Shape of ring
Uniform	Thickness of ring
Smaller diameter	Maximal diameter of ring
Smaller thickness	Maximal thickness of ring

**Table 6:** Evaluation of differential diagnosis efficacy for MUT BRAF and WT BRAF in E-GBM.

	E-GBM with MUT BRAF vs E-GBM with WT BRAF			
	AUC (95% CI)	Sensitivity	Specificity	Cutoff
Quantity of ring	0.878 (0.681-1.000)	0.923	0.833	0.500
Quantity of located lobe	0.801 (0.578-1.000)	0.769	0.833	0.500
Shape of ring	0.801 (0.578-1.000)	0.769	0.833	0.500
Thickness of ring	0.154 (0.000-0.328)	0.000	1.000	2.000
Maximal diameter of ring	0.167 (0.000-0.465)	1.000	0.167	2.895
Maximal thickness of ring	0.051 (0.000-0.159)	0.000	1.000	2.610
Quantity of ring + Quantity of located lobe	0.891 (0.715-1.000)	0.923	0.833	0.538
Quantity of ring + Shape of ring	0.962 (0.880-1.000)	0.923	0.833	0.625
Quantity of located lobe + Shape of ring	0.923 (0.794-1.000)	1.000	0.667	0.375
Quantity of ring + Quantity of located lobe + Shape of ring	0.962 (0.880-1.000)	0.923	0.833	0.625
Quantity of ring + Quantity of located lobe + Shape of ring + Thickness of ring +Maximal diameter of ring + Maximal thickness of ring	1.000 (1.000-1.000)	1.000	1.000	0.500

AUC, area under curve.

## Discussion

GBM is the most malignant tumor with poor prognosis in brain. The effect of routine treatments including operation, postoperative radiotherapy and chemotherapy is unsatisfied (20, 21). Recent studies have demonstrated that the mutation of BRAF in V600E was detected in GBM, especially frequently in E-GBM which is a new defined pathological subtype of GBM according to WHO 2016 criteria (22–27), and a histological pattern of “GBM, IDH-wildtype” in the WHO classification in 2021 (28). It was reported that the mutation of BRAF in V600E was closely related to E-GBM patients’ prognosis and some targeted therapies of MUT BRAF have already been used in clinical trials (18, 19). However, the mutation status of BRAF must be confirmed by postoperative pathological examinations. These processes often take a lot of time and the good opportunity for treating patients may be missed. Because the ring enhancement appearance in GBM’s MRI is special and intuitive, we considered that if this radiological examination could help us to predict the mutation status of BRAF preoperatively and guide timely targeted therapy. Lim-Fat et al. demonstrated that most GBMs with MUT BRAF were well circumscribed. Almost all tumors showed contrast-enhancing and tended to disseminate and migrate to subependymal or leptomeningeal at progression on MRI (29). In one case–control cohort, some radiographic features including well circumscribed borders, presence of large cysts with thin walls, and cortical involvement were reported closely related to GBMs with MUT BRAF (30). Ishi et al. further found that GBMs with MUT BRAF had radiological characteristics such as well circumscribed border, contrast-enhancing, large cystic component, mild perifocal edema, hemorrhagic onset and prior lesion on MRI (31).

MUT BRAF could activate MAPK signaling pathway, causing subsequent uncontrolled tumor cells’ proliferation (32, 33). Results of our study showed that most GBMs with MUT BRAF tend to be multiple and located in multiple lobes. This seemingly contradicted with the findings that the majority of MUT BRAF GBMs show a single lesion on MRI revealed by previous researches (29, 34). However, previous study demonstrated that the mutation of BRAF was more likely to occur in unstable microsatellite tumors in colorectal cancer (CRC), and half of CRC with MUT BRAF had metastasis (35). In addition, epithelial-mesenchymal transition (EMT) was found to influence GBM’s progression as well (36, 37). All these conclusions may explain why there were some cases with independent multiple ring enhancement lesions in this study. Compared to WT BRAF GBM, the shapes of ring enhancement in GBMs with MUT BRAF were more regular. It is known that most BRAF mutation of GBM happened in E-GBM. This special subtype had well circumscribed boundaries between tumor and surrounding tissues both in histopathological slides and MRI images. Besides, the intracranial growth space is limited and the heterogeneity of E-GBM cells is lower than that of conventional GBM cells. All these factors work together, easily making the shape of ring enhancement in GBM with MUT BRAF approximately round or oval. It is well known that fast-growing GBMs caused by MUT BRAF were short of enough nutrition and blood supply, so that they often contained intra-tumoral liquefactions such as cystic change, intra-tumoral necrosis and hemorrhage. Moreover, the histopathology of E-GBM showed the appearances of low-grade glioma whose thickness of ring enhancement was mostly uniform (25, 38). Thus, these factors may let the thickness of ring enhancement in GBM with MUT BRAF become more uniform than WT BRAF. For similar reasons, under the limited intracranial space and undernourished conditions, the maximal diameters of most GBMs with MUT BRAF were smaller than GBMs with WT BRAF. Taken together, the present study proposed several newly-defined radiological features of MUT BRAF GBM ring enhancement including more regular shapes, uniform thickness of wall and smaller maximal diameter, which was greatly different from past studies about radiological characteristics in BRAF mutant GBM (29–31).

**Figure 4:**
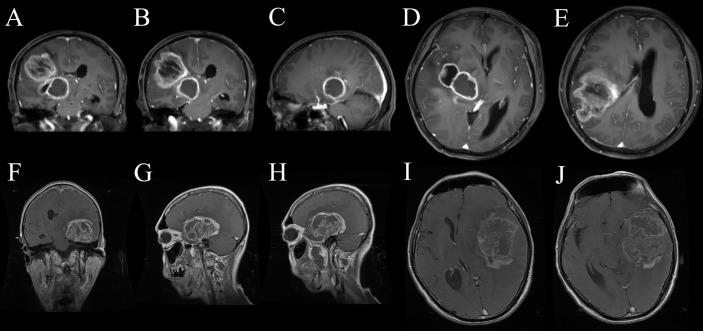
The appearances of ring enhancement on enhanced MRI of 1 GBM patient with MUT BRAF **(A–E)** and another one with WT BRAF **(F–J)**. The patient with MUT BRAF showed almost all the characteristic radiological appearances we proposed such as multiple ring enhancement appearances **(A)**, multiple lobes **(B)**, regular shape **(C)**, uniform thickness of ring **(D)** and small diameter of ring **(E)**. The patient with WT BRAF showed the opposite images such as single ring enhancement appearances **(F)**, single lobes **(G)**, irregular shape **(H)**, nonuniform thickness of ring **(I)** and relatively larger diameter of ring **(J)**.

The results of ROC curve analysis mean that the biological characteristics of GBM influenced by MUT BRAF were reflected in a series of imaging changes. The differential imaging manifestations would not appear alone. Comprehensive evaluation with multiple preoperative enhanced MRI characteristics may be useful to predict the mutation of BRAF in GBM. **Figure 4** showed the appearances of ring enhancement on MRI of 1 patient with MUT BRAF and 1 patient with WT BRAF. The images of former patient showed almost all the characteristic radiological appearances we proposed above about MUT BRAF GBM. In addition, three MRI manifestations including quantity of ring, shape of ring and maximal diameter of ring were used as the nomogram score to build a model which has a good predictive ability of BRAF’s mutant status in this study.

E-GBM is a new variant of GBM which has been provisionally added to the 2016 version of classification of GBM. Previous literatures demonstrated that more than half E-GBMs were with BRAF mutation, while the mutation of BRAF rarely happened in conventional GBMs (22–27). The targeted therapy using BRAF kinase inhibitor such as dabrafenib and vemurafenib has been reported to improve the prognosis of E-GBM patient with MUT BRAF (18, 19) despite the rarity of data about the efficacy of BRAF inhibitor for treatment of patients with GBM presently. It is worth noting that the mutation rate of BRAF in GBM was higher in our study compared with that reported by previous literatures, 6% (33, 39). The reasons were as follows: 1) The high BRAF mutation rate of GBM in our study may be resulted from a high proportion of E-GBMs whose BRAF mutation rate was more than 50%; 2) Differences in human species may cause the bias about the BRAF mutated proportion in GBM; 3) Only 44 cases and 2 departments could not sufficiently reflect the truth. Larger samples are needed in further study. To be noted, the mutation rate of BRAF in E-GBM was 68.42% (13/19) which was generally consistent with past research results. In other words, the above results could be applied to differential diagnosis of the mutation status of BRAF in E-GBM, so that they could provide potential reference for targeted therapy application in clinic. Thus, the additional exploration was carried out between E-GBM with MUT BRAF and with WT BRAF. As expected, the re-analysis showed almost the same results. Besides the 5 different variables mentioned above in GBM with MUT BRAF and with WT BRAF, the maximal thickness of ring was significantly thinner in MUT BRAF group than in WT BRAF group which could also be explained by our previous deduction. The ROC curve analysis was performed as well. All of these results indicated that preoperative characteristic radiological appearances could play a certain role in predicting whether BRAF mutated or not in E-GBM.

This is not the first study to predict BRAF mutation status of tumors by radiographic features on MRI. Yue et al (40). proposed a potential diagnostic criterion for BRAF-mutated craniopharyngiomas which was composed of several MRI characteristics such as suprasellar location, spherical shape, predominantly solid component, homogeneous enhancement, and pituitary stalk thickening. This significant research has inspired us to initiate a study to predict BRAF mutation status in GBM based on MRI features, guiding timely preoperative targeted therapy. However, there were some significant differences between our research and Yue’s study. 1) the object of study by Yue et al. was craniopharyngiomas, while our study was GBM; 2) our research paid more attention to ring enhancement appearance on MRI, which was a characteristic performance of GBM; 3) given that the location and components of craniopharyngioma were special, the study included some important radiographic characteristics such as encasement of internal carotid artery, suprasellar intrusion, and predominantly solid or cysts; 4) our study constructed a nomogram, hoping to offer a potential reference for preoperative treatment of BRAF mutant GBM with BRAF inhibitors.

### Study limitations

However, this is still a preliminary study and there are a few limitations. First, the analysis of GBM gene mutations in our hospital just started from 2016, the genetic mutation information of previous GBM samples were missing. Information at present consisted of a relatively few cases and lacked enough different pathological subtypes of GBM. For example, most cases with MUT BRAF came from E-GBMs. Only 1 gliosarcoma and 1 conventional GBM were with MUT BRAF. Both the morbidity of E-GBM and the mutation rate of BRAF in this study were extremely higher compared to previous reports. This must be a coincidence, and it was impossible to compare it with other pathological subtypes of GBMs when their case numbers differed largely. Except that, this situation even made the combination of all the 6 statistically different variables’ AUC become 1.000 which is probably a false positive result caused by selection bias. The nomogram for predicting the mutant status of BRAF in E-GBM also cannot be achieved currently due to the small number of cases. Thus, to avoid these problems, larger and multi-center studies are needed to confirm our preliminary results. Second, the results were inevitably influenced by some human factors as well such as the work habits of neuroradiologists, the selection of imaging levels when making judgements and the controversial images. Third, other possible influence factors are still unknown. All these influence factors need to be eliminated in further studies. Hence, the relationship between BRAF mutation status and preoperative enhanced MRI appearances in GBMs should be studied more deeply.

## Conclusions

In conclusion, we found that characteristic ring enhancement appearances of GBM may play a critical role in predicting BRAF mutation status preoperatively, especially in E-GBM. The prediction can be established only when at least 3 features are included, such as quantity of ring, quantity of located lobe and shape of ring. Further study with larger cases may provide more reliable evidences to guide the pretreatment of targeted therapy for GBM patients with MUT BRAF in the future.

## Data availability statement

The data analyzed in this study is subject to the following licenses/restrictions: This article only involves clinical data, but these data contain the patient’s personal privacy and need to be kept confidential. Requests to access these datasets should be directed to chenming@xinhuamed.com.cn.

## Ethics statement

The studies involving human participants were reviewed and approved by Ethics Committee of Xinhua Hospital, School of Medicine, Shanghai Jiaotong University. The patients/participants provided their written informed consent to participate in this study.

## Author contributions

MC, XW, and SL designed and supervised the project. XC, ZC, BC, and MT collected clinical data and performed data analysis. XC and MC wrote and revised the manuscript. All authors contributed to the article and approved the submitted version.

## Funding

This work was supported by the National Natural Science Foundation of China (grant number: 81902521), Shanghai Sailing Program (grant number: 19YF1432800), Research Project of Xinhua Hospital (grant number: XH1936), Doctoral Research Fund of the First Affiliated Hospital of USTC (grant number: RC2021121) and WenZhou Science and Technology Bureau Project (grant number Y20190143).

## Conflict of interest

The authors declare that the research was conducted in the absence of any commercial or financial relationships that could be construed as a potential conflict of interest.

## Publisher’s note

All claims expressed in this article are solely those of the authors and do not necessarily represent those of their affiliated organizations, or those of the publisher, the editors and the reviewers. Any product that may be evaluated in this article, or claim that may be made by its manufacturer, is not guaranteed or endorsed by the publisher.
