# Supplementary material for: Prediction of BRAF mutation status in glioblastoma multiforme by preoperative ring enhancement appearances on MRI
Source: Front Oncol. 2022 Aug 8;12:937345. doi: 10.3389/fonc.2022.937345 (PMC9393738; doi:10.3389/fonc.2022.937345)
Supplement: Supplementary file 1 [file Table_1.docx]

**Supplementary table 1. Impacts of MRI manifestations on BRAF’s mutant status in multivariate model**

|  | Unadjusted | |  | Adjusted | |
| --- | --- | --- | --- | --- | --- |
|  | OR (95% CI) | *P* value |  | OR (95% CI) | *P* value |
| Quantity of ring | 2.741 (1.102-6.816) | 0.030 |  | 0.278 (0.089-0.861) | 0.027 |
| Quantity of located lobe | 1.971 (0.809-4.805) | 0.135 |  | 0.706 (0.222-2.244) | 0.555 |
| Shape of ring | 2.700 (1.059-6.883) | 0.037 |  | 0.231 (0.065-0.815) | 0.023 |
| Thickness of ring | 0.750 (0.307-1.835) | 0.529 |  | 0.540 (0.162-1.799) | 0.316 |
| Maximal diameter of ring | 0.385 (0.227-0.655) | ＜0.001 |  | 3.343 (1.787-6.255) | ＜0.001 |
